# Mathematical models for biomarker calculation of drug-induced liver injury in humans and experimental models based on gadoxetate enhanced magnetic resonance imaging

**DOI:** 10.1371/journal.pone.0279168

**Published:** 2023-01-06

**Authors:** Markus Karlsson, Christian Simonsson, Nils Dahlström, Gunnar Cedersund, Peter Lundberg

**Affiliations:** 1 Department of Health, Medicine and Caring Sciences, Linköping University, Linköping, Sweden; 2 Center for Medical Image Science and Visualization, Linköping University, Linköping, Sweden; 3 Department of Biomedical Engineering, Linköping University, Linköping, Sweden; 4 Department of Radiology, Department of Health, Medicine and Caring Sciences, Linköping University, Linköping, Sweden; 5 Department of Radiation Physics, Department of Health, Medicine and Caring Sciences, Linköping University, Linköping, Sweden; Heidelberg University, GERMANY

## Abstract

**Background:**

Drug induced liver injury (DILI) is a major concern when developing new drugs. A promising biomarker for DILI is the hepatic uptake rate of the contrast agent gadoxetate. This rate can be estimated using a novel approach combining magnetic resonance imaging and mathematical modeling. However, previous work has used different mathematical models to describe liver function in humans or rats, and no comparative study has assessed which model is most optimal to use, or focused on possible translatability between the two species.

**Aims:**

Our aim was therefore to do a comparison and assessment of models for DILI biomarker assessment, and to develop a conceptual basis for a translational framework between the species.

**Methods and results:**

We first established which of the available pharmacokinetic models to use by identifying the most simple and identifiable model that can describe data from both human and rats. We then developed an extension of this model for how to estimate the effects of a hepatotoxic drug in rats. Finally, we illustrated how such a framework could be useful for drug dosage selection, and how it potentially can be applied in personalized treatments designed to avoid DILI.

**Conclusion:**

Our analysis provides clear guidelines of which mathematical model to use for model-based assessment of biomarkers for liver function, and it also suggests a hypothetical path to a translational framework for DILI.

## Introduction

Drug-induced liver injury (DILI) is a potentially serious condition which can cause acute liver failure, and even require transplantation [1]. Therefore, DILI is a major concern when developing new drugs [2]. The exact mechanisms and proclivity for DILI are unknown and probably varies between patients; presently, only non-specific biomarkers are measured to address both these issues. The most common biomarker for DILI is plasma concentrations of alanine aminotransferase (ALT) [3]. However, ALT is neither specific, nor is it an actual measure of liver function; rather ALT is an indicator of past liver injury [4]. Another biomarker, total bilirubin, is more specific, but is only sensitive in late stages of DILI [1].

**Fig 1:**
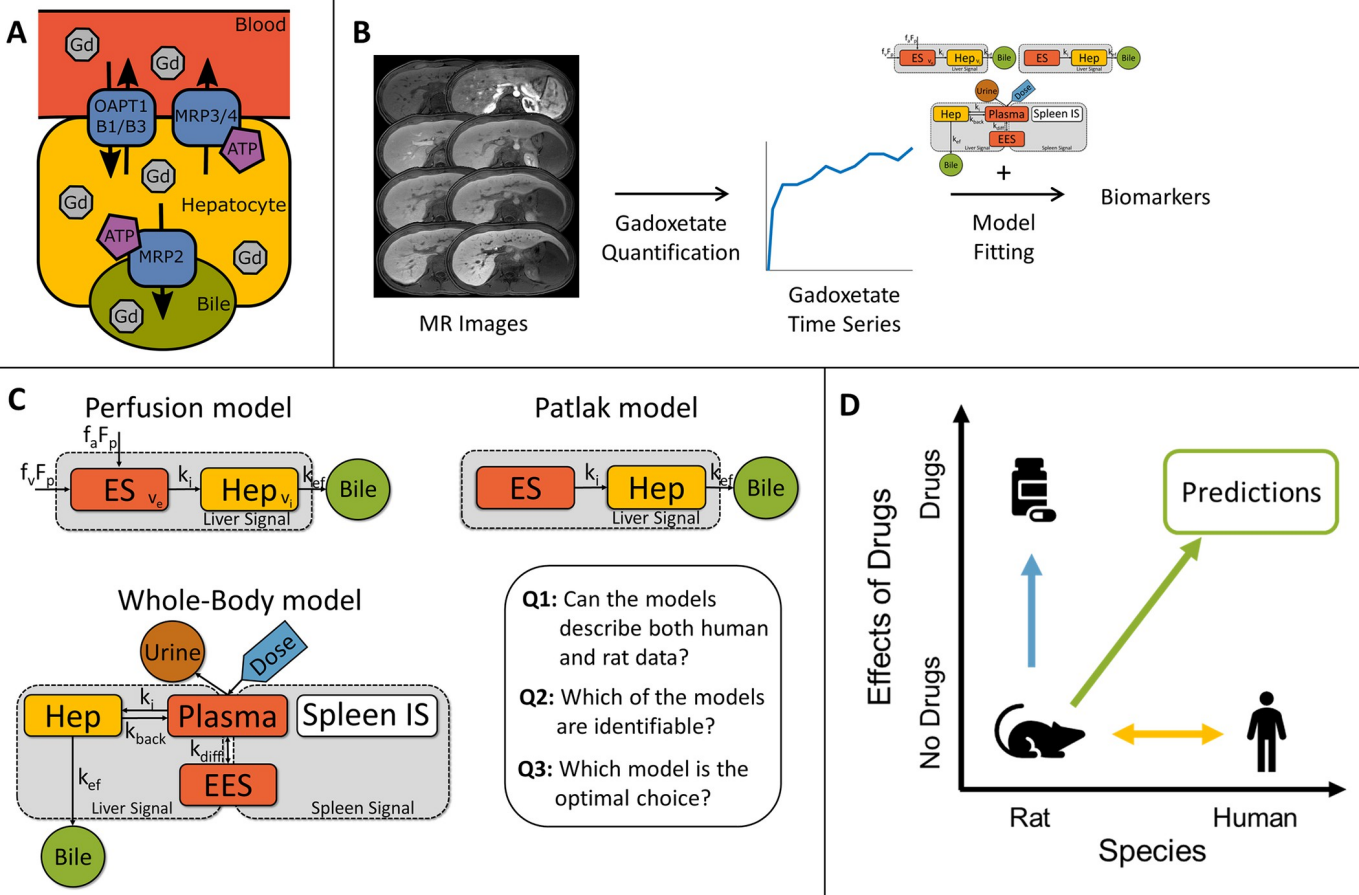
Biological background and modelling workflow. **(A) Model.** Gadoxetate, a gadolinium ion (Gd^3+^)-containing MRI-contrast agent, is taken up by hepatocytes via the OATP1B1/B3 transport proteins. After uptake, gadoxetate is actively excreted into the bile via the MRP2 transport proteins. Gadoxetate can also to some extent be transported back to the blood via MRP3/4 transporters. **(B) Modelling workflow.** After acquiring MR images before and after gadoxetate injection, the images are processed to get timeseries of gadoxetate concentrations. Finally, the timeseries are combined with pharmacokinetic models in order to estimate the gadoxetate transport rates, which are used as biomarkers for liver function.**(C) Model structures for the Patlak, Perfusion, and Whole-Body models.** Abbreviations: Hep, hepatocyte compartment; ES, liver extracellular compartment; Plasma, blood plasma compartment; EES, extracellular extravascular compartment; Spleen IS, intracellular spleen compartment; k_i_, hepatic uptake rate; k_ef_, hepatic efflux rate; k_back_, Gadoxetate transport rate back to the blood; F_p_, total blood flow to the liver; f_a_, arterial flow fraction; f_v_, portal venous flow fraction (i.e., 1-f_a_). **(D) Purpose of the study.** Previous research has focused on how liver function and gadoxetate pharmacokinetics can be described in humans as well as in rats (yellow arrow; see Figs 3–5). Other research has also studied how liver function can be affected by drugs (blue arrow, see Fig 5). However, the clinically interesting question of how a given drug affects liver function in humans is much more difficult to answer. We therefore here introduce a translational modelling framework in order to predict how a given drug can affect liver function in humans (green arrow, see Fig 6).

An alternative path for measuring liver function involves dynamic contrast-enhanced magnetic resonance imaging (DCE-MRI) using gadoxetate (Gd-EOB-DTPA, Primovist®, Eovist®, Bayer Healthcare Pharmaceuticals, Berlin, Germany), which is a liver-specific contrast agent [5]. In humans (Fig 1A), gadoxetate is taken up by hepatocytes, via the organic anion-transporting polypeptide 1 (OATP1) transporters and excreted into bile via the multidrug resistance-associated protein 2 (MRP2). Apart from being routinely used in the clinic to detect focal lesions in the liver [6], gadoxetate-MRI has also shown promising results as a measure of liver function, as analysis of the gadoxetate pharmacokinetics provides direct information about the functions of both OATP1 and MRP2 transporters. These transporters constitute central parts of the livers´ ability to clear substances such as bile acids and toxins from the blood, which is one of the basic functions of the liver [7, 8].

An array of different methods has been introduced to estimate gadoxetate pharmacokinetics in humans. The simplest methods involve measuring ratios of signal intensities in MR-images, before and at a chosen time-point after injection [9, 10]. While such methods are easy to implement in a clinical setting, they are unable to quantify the actual gadoxetate transport rates using such simplistic procedures; specifically, it is not possible to separate the effects of uptake from excretion. A more sophisticated approach is to perform pharmacokinetic modelling, which can provide estimates of transport rates (Fig 1B). Unlike the signal ratio methods, such modelling typically requires time series of gadoxetate concentrations, and thus multiple images need to be acquired. Several different modelling approaches have been introduced, see Discussion. Methods that vary with respect to both model complexity and structure as well as what type of input data that are required.

Most previous work on liver function and gadoxetate have either focused on describing normal or pathological liver function in either humans or rats, but rarely on how to translate quantitative findings between species (Fig 1D; yellow). Similarly, work has been done on how drugs affect liver function in rats and how this can be investigated with gadoxetate, but models describing such effects has not been employed (Fig 1D; blue). Finally, it also remains to develop protocols to allow for the translation of pharmacological effects observed in rats to realistic clinical situations and personalized treatments in individual human subjects (Fig 1D; green).

In this study, we aimed to investigate three different mathematical models with respect to three main questions (Fig 1): Q1: *Can the models describe both human and rat data*?; Q2: *Which of the models are identifiable*?; Q3: *Which model is the optimal choice*? Our second aim was then to select and expand the most suitable model to allow for translation of experimentally observed pharmacological effects, from rats to humans (Fig 6). Finally, we attempted to illustrate how this capability in principle could be useful for dosage selection, with a potential focus on personalized treatments with a specific perspective on how to avoid DILI.

## Materials and methods

### Human subjects

This study included 35 patients, from a previously described prospective cohort [11], undergoing a liver biopsy due to suspected chronic liver disease. The original cohort included 91 patients. Of these patients, 59 had images acquired every ten minutes whereas the other 36 patients had images acquired four times as often. Only the last 36 patients were included in this study. Furthermore, one of the 36 patients was excluded because the examination had been aborted. The human study was approved by the Swedish Ethical Review Authority (Dnr M72-07, NILB) and informed consent was obtained from the subjects [11].

### Rats

The study also included data from rats, previously described in [12]. Briefly, 30 male Han Wistar rats were administrated chemokine antagonist (CKA) [12], which inhibits biliary transporter activity. The CKA was six different doses (0, 20, 50, 200, or 2000 mg/kg) 30 minutes before imaging.

### Human imaging and post-processing

The human image acquisition and post-processing methods have previously been described in [11]. In short, DCE-MRI was performed using a 1.5 T MR-scanner (Philips), acquiring two-point Dixon 3D images, before and after a bolus injection of gadoxetate. The post-injection images included arterial and portal venous phases, an equilibrium phase at 3 minutes, and late phases up to 30 minutes post-injection. In between, images were acquired every two or three minutes (*i*.*e*. 3, 5, 8, 10, etc). Seven regions of interests (ROIs) were placed in the liver, three ROIs were placed in the spleen, one ROI was placed in the aorta and the portal vein. The average signal intensities from the ROIs were normalized and converted into changes in R1 relaxation rates [13], which in turn was converted into gadoxetate concentrations assuming a relaxivity of 6.9 s^-1^mM^-1^ [14] in the blood and spleen and 10.7 s^-1^mM^-1^ [15] in the liver at 1.5 T.

### Rat imaging and post-processing

The rats images were acquired using a 4.7 T MR-scanner [12]. Before DCE-MRI, baseline R1 of the liver and spleen was estimated using an inversion recovery FISP sequence. Afterwards, DCE-MRI was performed using a coronal IntraGate FLASH sequence. Images were then acquired every minute for 60 minutes. ROIs were drawn covering the whole liver and spleen and the signal intensities were converted into changes in R1, which was converted into gadoxetate concentrations assuming a relaxivity in the liver and spleen of 5.9 s^-1^mM^-1^ at 4.7 T. More details can be found in [12].

### Modeling

We used three different model to describe the data, the Patlak model, the perfusion model, and the whole-body model. The details of these models and the cost functions used are described in some detail in the supplemental material (section 1 and 2). All necessary Matlab code and model-files can be found in our GitHub Repository (github.com/chrsi30/MMDILI) with back-up on Zenodo (DOI: 10.5281/zenodo.6671138). This constitutes the minimal data set that is required to reach the conclusions drawn in the report.

### Profile likelihood analysis

Identifiability of model parameters was investigated using the profile likelihood approach [16, 17]. In a profile likelihood analysis, the parameters are increased (or decreased) in a stepwise fashion, while optimizing all other parameters. As the value of the parameter is stepped further away from optimum, the estimated cost will increase. This is done until the cost function reaches a predetermined cutoff value. The cutoff was chosen as the optimal cost plus the 95^th^ percentile of the χ^2^-distribution with one degree of freedom. In other words, we use a p-value of 0.05, meaning a 95% confidence interval, thus the true parameters should lie within the confidence interval.

### Dose-response modelling

To quantitatively estimate the effects of the drug on the pharmacokinetic parameter *k*_*x*_ in rats j, the parameters for each rat was modelled as containing two parts:

$$k_{x,j}={min}_{x}+\frac{{max}_{x}-{min}_{x}}{1+{\left(\frac{{IC50}_{x}}{{dose}_{j}}\right)}^{{Hill}_{x}}}+p_{x,j}.$$ [1]

The first part is a dose-response part describing the expected mean parameter values for each rat, given a dose of the drug, where *min*_*x*_ is the minimum value of *k*_*x*_, *max*_*x*_ is the maximum value of *k*_*x*_; *IC*_*50x*_ is the dose of the drug which give half the value of *k*_*x*_, *Hill*_*x*_ is the Hill coefficient for *k*_*x*_ and *dose*_*j*_ is the drug dose given to *rat j*. The second part, *p*_*x*,*j*_, describes how the individual value of parameter *k*_*x*_ for rat *j* deviates from the expected parameter value.

All parameters were jointly estimated using a nonlinear mixed-effects-modelling (NLME) approach [18]. The dose-response parameters, *min*_*x*_, *max*_*x*_, *IC*_*50x*_, and *Hill*_*x*_, were estimated as only fixed effects, *i*.*e*. they were the same for all rats. The remaining parameter, *p*_*x*,*j*_, was estimated as having only a random effect, *i*.*e*. the parameter varied between the rats. The NLME-parameter estimation was performed using the built-in Matlab function *nlmefitsa* [19].

### Human liver function prediction

The magnitude of the possible effect of the drug, given and measured in the rats, would have on humans was illustrated by simulating the gadoxetate uptake in the liver at different doses of the drug. The parameters for the simulation was calculated using Eq.13 with *IC*_*50x*_ and *Hill*_*x*_ assumed to have the same value as determined using rat data and the maximum and minimum value for each patient calculated according to:

$${max}_{x,human}=k_{x,human}$$ [2]

$${min}_{x,human}={max}_{x,human}\frac{{min}_{x,rat}}{{max}_{x,rat}}$$ [3]

where *max*_*x*,*human*_ and *min*_*x*,*human*_ are maximum and minimum-values respectively, used for simulation, *k*_*x*,*human*_ is the parameter value originally estimated for the human patient, and *max*_*x*,*rat*_ and *min*_*x*,*rat*_ are maximum and minimum-values estimated for the rats.

## Results

### All models can describe data from humans

**Fig 2:**
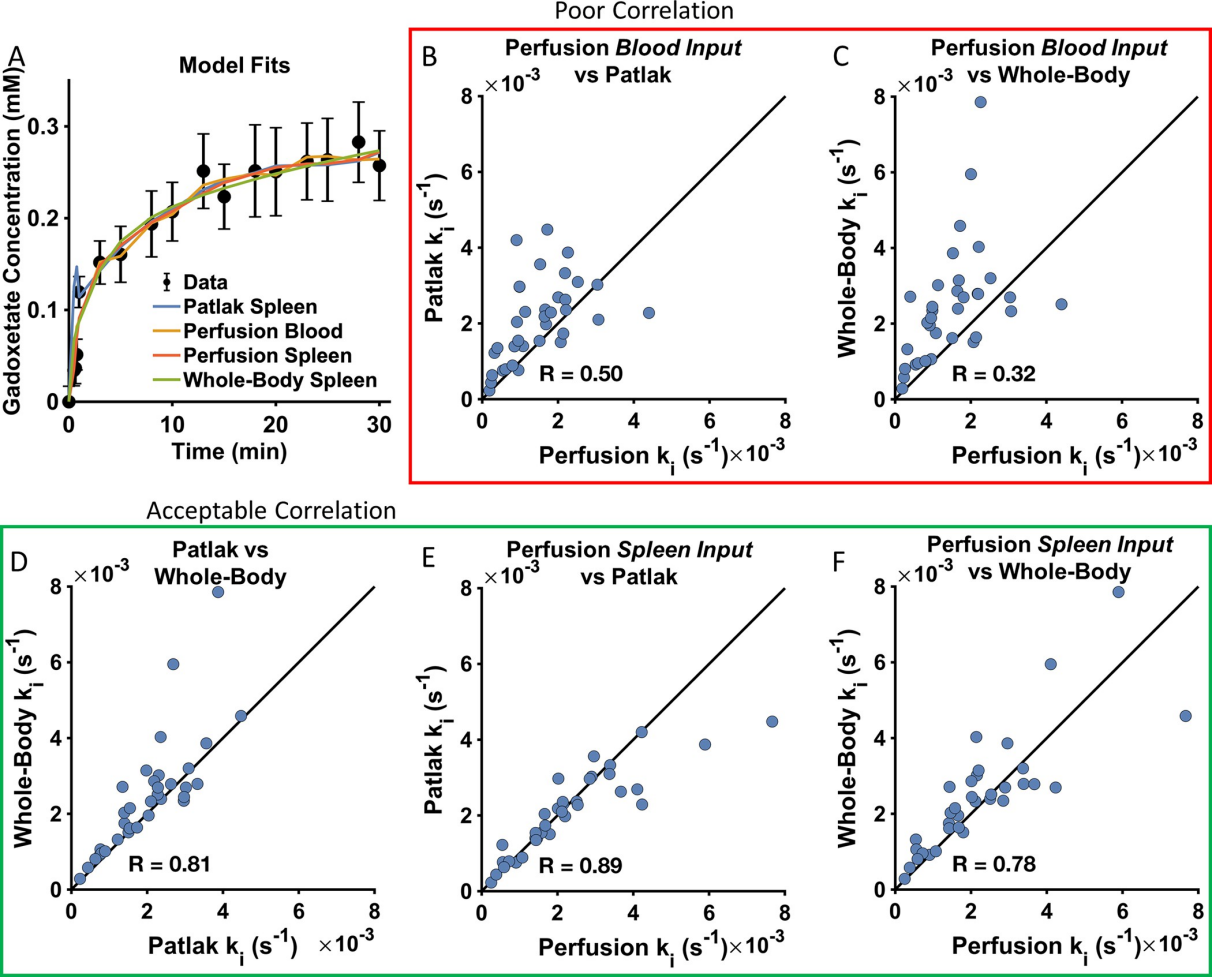
All models can describe human data and gives the same uptake rates with spleen as input. **(A)** Example of how well the models fitted to the data from the liver from a typical patient. **(B-F)** Correlations between the gadoxetate uptake rates, k_i_, determined using different pairs of models. **(B)** the Perfusion vs. Patlak-model, with input from blood; **(C)** the Perfusion- vs. the Whole-body-model, with input from blood; **(D)** the Patlak vs. Whole-Body models, with input from blood; **(E)** the Perfusion model vs. the Patlak model, with input from the spleen, **(F)** the Perfusion vs. the Whole-Body model, with input from the spleen. Black lines in **(B-F)** show the unity line.

All three pharmacokinetic models (Fig 1) were fitted to human data from patients with suspected chronic liver disease [11]. The liver concentration of gadoxetate of one patient is shown in Fig 2A, together with liver concentrations that were simulated using the three models. The Perfusion model was fitted using either the blood or spleen signals as inputs. The figure shows that all three models were able to fit to the data. When testing for goodness-of-fit, all 35 patients passed a χ^2^ test with the Patlak model, the Whole-Body model, and the Perfusion model. In this test, the Perfusion model used blood as input signal. When using the perfusion model with spleen as input signal, 29 patients (83%) passed the χ^2^ test.

Fig 2B and 2C shows the uptake rate estimated using the perfusion model (using blood as input) correlated with the uptake rates estimated using the Patlak and Whole-Body models. The two figures show a low correlation, (R ≤ 0.5), but the correlation was higher when comparing the whole-body and Patlak models (both using spleen as input) (Fig 2D; R = 0.81). To investigate if the discrepancies in Fig 2B and 2C was due to differences in model structure or due to differences in input signal, Fig 2E and 2F shows correlation using the perfusion model with the spleen as input. In this case, the correlation was larger and also similar to the correlation between the Patlak and Whole-Body models.

In summary, all models could describe human data and resulted in the same uptake rate parameters, as long as the same input was used.

### All models can describe data from rats

**Fig 3:**
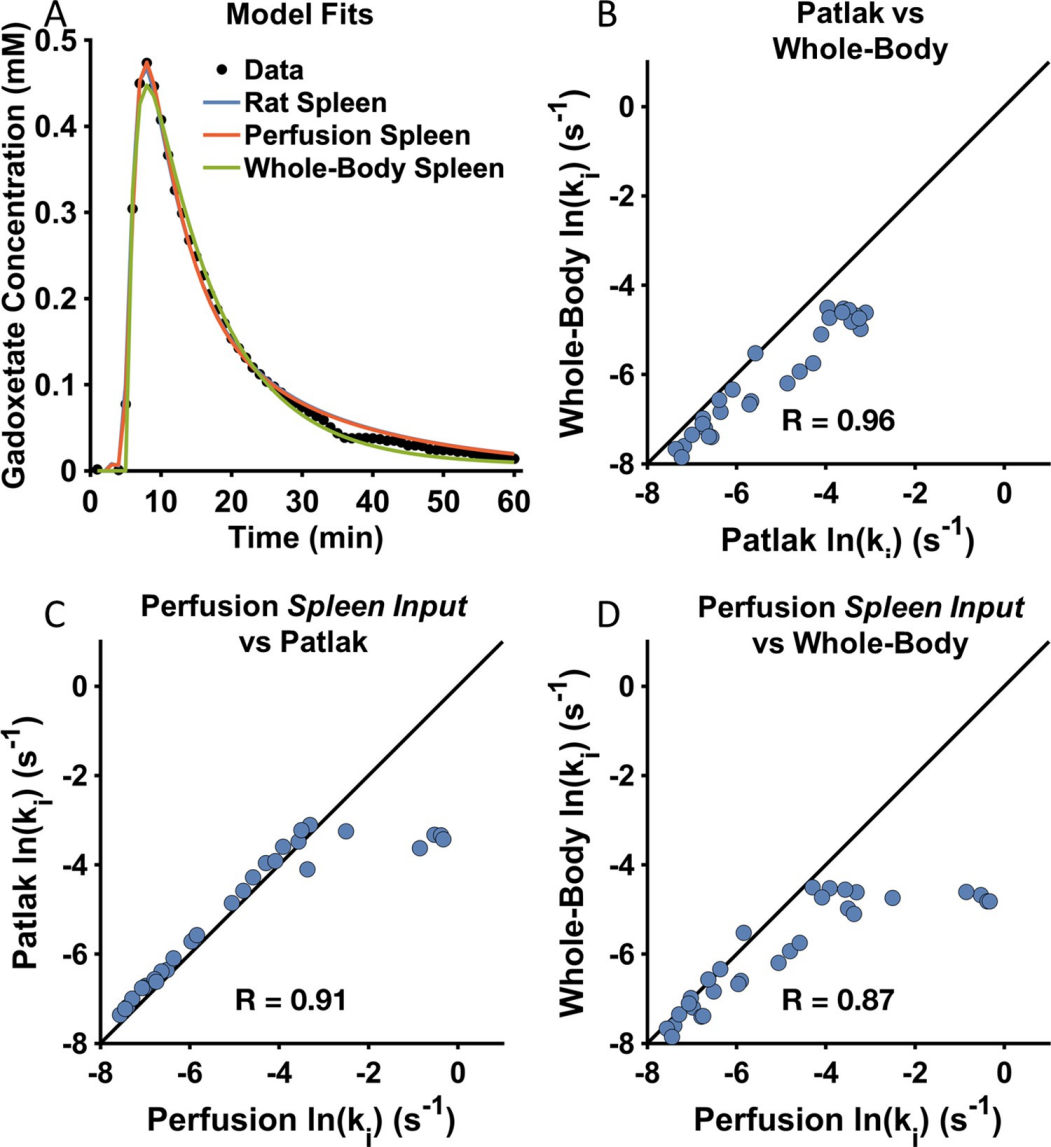
All models can describe rat data. (A) Example of how the models fit to the data from the liver from one of the rats. (B-D) Correlations between the logarithm of the gadoxetate uptake rate, k_i_, estimated using (B) the Patlak and Whole-Body models, (C) the Perfusion model, with input from the spleen, and the Patlak model, (D) the Perfusion model, with input from the spleen, and the Whole-Body model.

The models were also fitted to data from rats treated with different doses of a chemokine antagonist (CKA), which causes DILI by inhibiting the function of both OATP1 and MRP2 [12]. Fig 3A shows the gadoxetate concentration in the liver from one of the rats together with fitted simulation from the three models. Note that a larger and more complete section of the uptake curve was observed in rats compared to humans. However, only input data from the spleen (not blood) was available from the rats. The figure shows that all three models were able to fit to the data. Fig 3B–3D shows the correlations between the uptake rates estimated using the different models.

The three figures show that the correlations were similarly large when comparing all three methods, although the whole-body model did underestimate the uptake rate (Fig 3B). The correlation between the perfusion and Patlak models was excellent although the Perfusion model slightly overestimated the highest uptake rates (Fig 3C).

### The Patlak model provided the most well-defined uptake rates

As all three model structures can fit to data from both humans and rats, and there were satisfactory correlations between the estimated uptake rates. For that reason, we wished to investigate which model is the optimal for determining uptake rates. To this end, we performed a profile likelihood analysis, which provided the uncertainty of the uptake rate [17]. Briefly, a profile likelihood estimates the uncertainty of a given model parameter by stepping that parameter through a suitable range, while optimizing over all other parameters. This is repeated, with the parameter of interest being stepped further and further away from optimum, until the optimized cost is higher than the optimal cost plus the 95^th^ percentile of the χ^2^-distribution with one degree of freedom.

**Fig 4:**
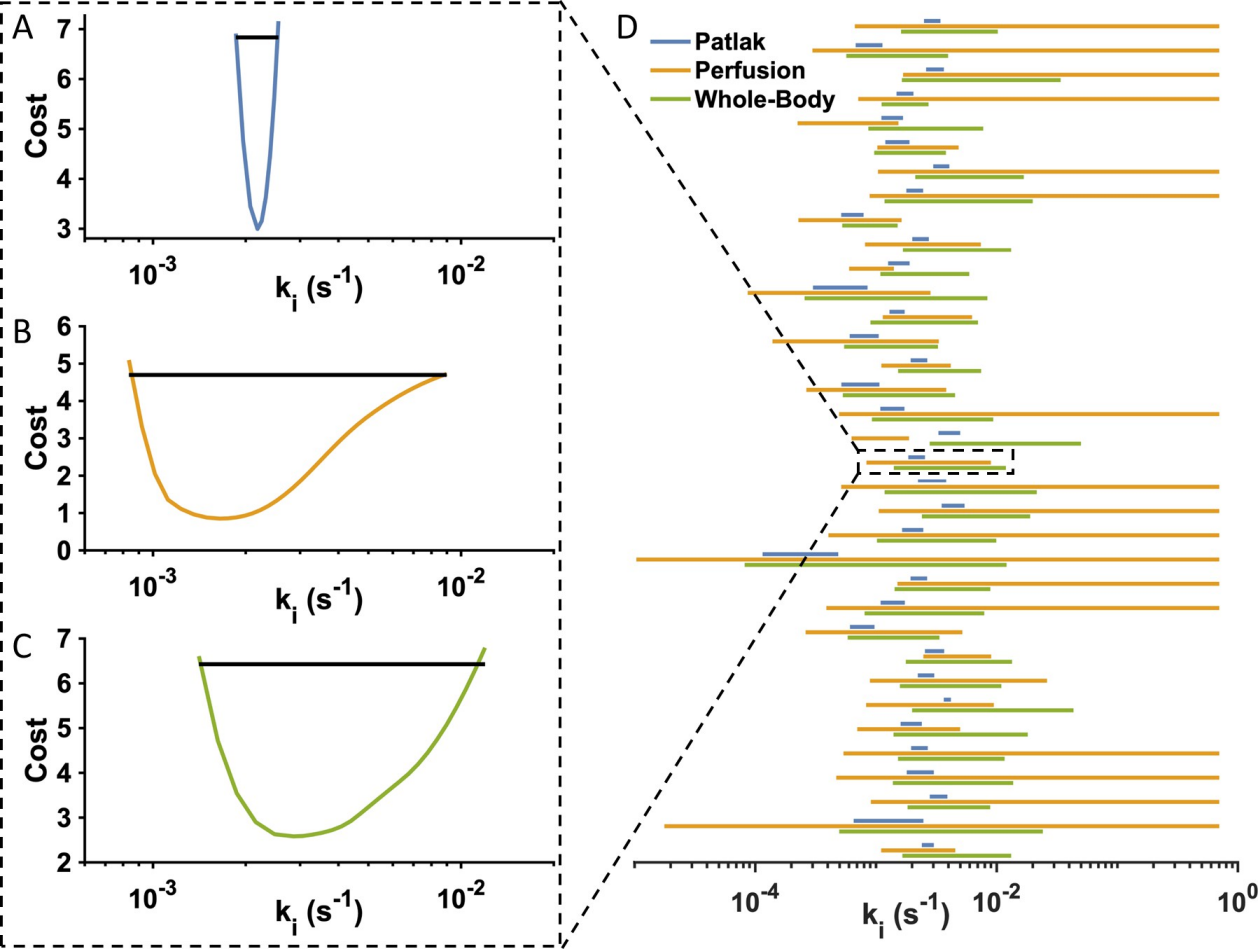
Likelihood profiles show that the Patlak model has the most well-defined uptake rates. The figure shows likelihood profiles of the gadoxetate uptake rate, k_i_, estimated using data from the same human patient and (A) Patlak model, (B) Perfusion model, and (C) Whole-Body model. Note, the same x-axis scaling in all panels. (D) shows the profile likelihood confidence intervals for all three models and all 35 individual patients. Note the large variation of k_i_ in the individual subjects. The dashed box represents the average patient for which three likelihood profiles are shown in panels (A-C).

Fig 4A–4C shows the likelihood profiles of the uptake rate for one typical human patient, determined using all three models. The figure clearly shows a narrower and more well-defined confidence interval for the Patlak model (Fig 4A–4C). Moreover, Fig 4D, which shows the confidence intervals for all 35 patients, indicates that the Patlak model (blue) systematically had the tightest (best) confidence intervals. This is not entirely unexpected, since the Patlak model is the model characterized by least complexity. In summary, the Patlak model fitted to both human and rat data equally well as the two alternative models. Although it is less mechanistic, the more well-defined uptake rates inferred that from a mathematical point of view it is the preferred model. Therefore, we only considered this model in the following analysis.

### Development of a dose-response model using rats

**Fig 5:**
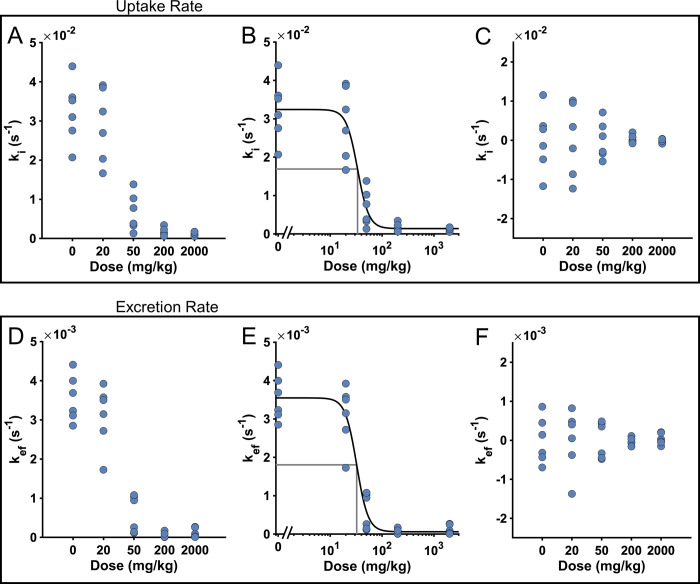
Dose-response of the drug in rats. **(A, D)** show the estimated k_i_ and k_ef_ grouped by drug dose. (B, E) show dose-response plots for k_i_ and k_ef_, respectively. The black curve is the dose response function and the gray lines shows the IC_50_ point. **(C, F)** show the variation of each parameter for each rat, i.e., how much the individual parameter values deviates from the value expected by the dose-response function.

An estimate of the effect of an inhibitor of biliary transport activity (chemokine antagonist (CKA) 1-(4-chloro-3-trifluoromethyl-benzyl)-5-hydroxy-1-H-indole-2-carboxylic acid) given to rats has been reported by Ulloa and co-workers [12]. In this work we have determined the pharmacokinetic parameters in the Patlak model for each individual rat, and Fig 5A and 5D shows the uptake rate, k_i_, and the excretion rate, k_ef_, grouped by the drug dose given to each rat. The figure shows a clear relationship between the drug and the estimated pharmacokinetic parameters.

This association between effector dose and inhibition creates the possibility to utilize a nonlinear mixed-effects (NLME) approach (see Materials and Methods), and the uptake rates for all rats were determined simultaneously in a single procedure. In addition, the range of drug doses affected the pharmacokinetic parameters in a dose dependent manner, determined by a dose response function. The specific parameters of the dose response function were then determined in combination with the array of pharmacokinetic parameters. Fig 5B and 5E shows these dose-response plots for k_i_ and k_ef_, respectively, including the estimated pharmacokinetic parameters. The estimated NLME parameters for the dose response curve are also shown in Table 1A. Finally, Fig 5C and 5F shows the individual variation for each individual rat (uptake and excretion respectively), *i*.*e*. the individual parameter subtracted from the dose response curve. It is evident that our dose-response model captures all dose dependencies, as the normalized individual parameters for all rats varies evenly around zero. In summary, we have developed a new NLME model describing how a specific drug impacts the uptake and excretion rates in rats.

**Table 1:** (A) Dose-response parameters. (B) Illustration of suggested translational drug dose in humans.

A.	Max (s^-1^)	Min (s^-1^)	IC50 (mg/kg)	Hill-Coefficient
k_i_	3.2x10^-2^	1.4x10^-3^	33.7	-3.9
k_ef_	3.5x10^-3^	6.1x10^-5^	32.4	-3.9
B.	Current Uptake Rate	Maximal Allowed Dose	Minimal Required Dose	Decision
P1	4.1 ms^-1^	44 mg/g	28 mg/kg	OK to use
P2	2.5 ms^-1^	35 mg/kg	28 mg/kg	OK to use
P3	1.4 ms^-1^	21 mg/kg	28 mg/kg	Need another drug

### Translating the NLME model to humans and personalized treatment design

**Fig 6:**
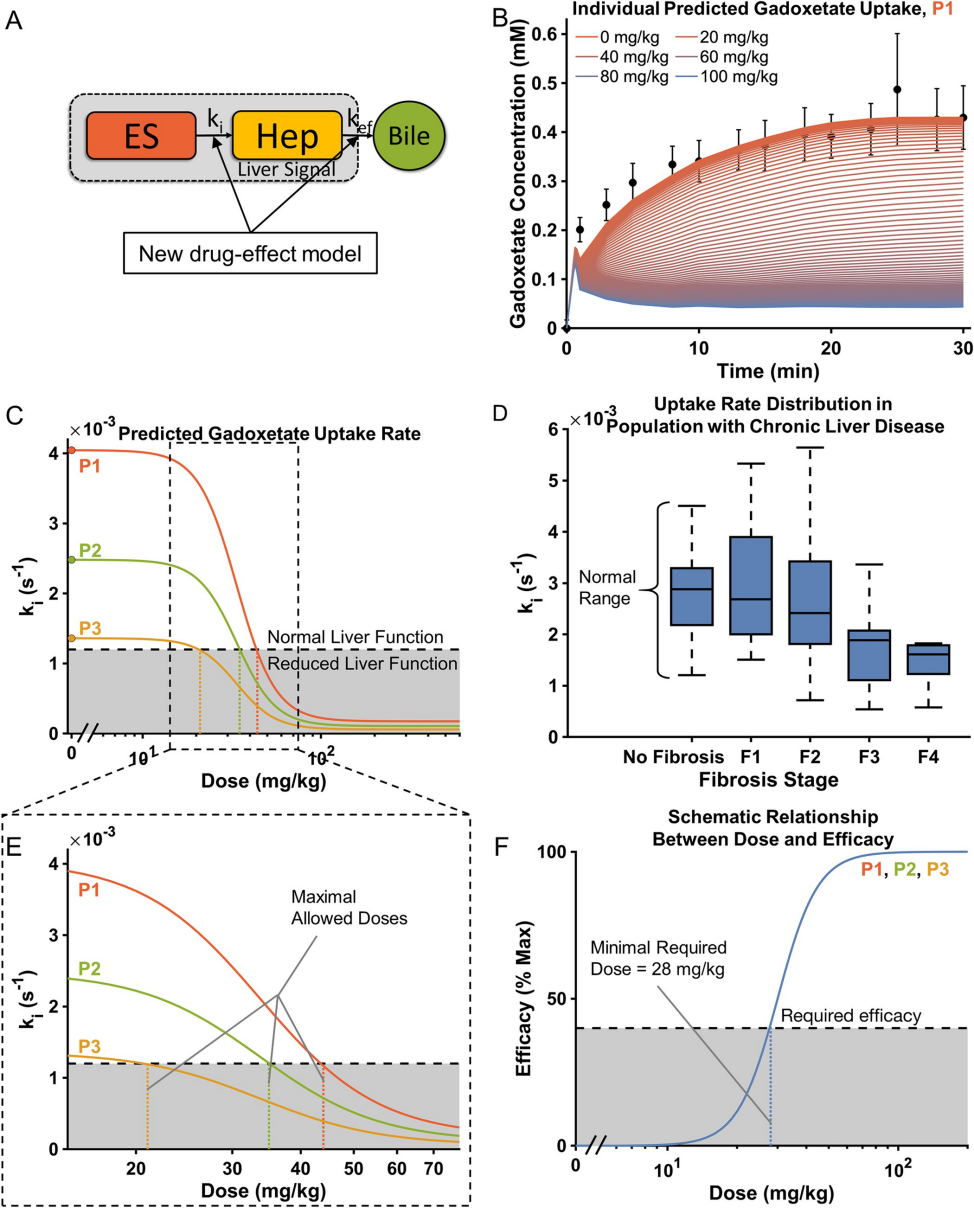
Illustration of how the modelling framework can be used to predict liver function. **(A)** The extended Patlak model, now including the effects of the drug on the transport rates. **(B)** The panel shows an example of how the uptake of gadoxetate would change if one of the human patients would be given the same drug as the rats, assuming that the translational dose-response of the drug is the same in both humans and rats (which may, or may not, be correct). The different colors of the lines correspond to different doses of the drug. **(C, E)** Examples of a dose-response of the uptake rate for three different individuals, with different baseline uptake rates. The panels show that there are individual doses at which the uptake rates would fall below the range of normal liver function. Thus, resulting in a different maximal allowed dose for each patient. **(D)** The range of normal liver function is taken from (20) where normal function is defined as the uptake rate in patients without fibrosis. **(F)** A schematic illustration of how the efficacy of a drug is related to the translational dose. Typically, there is also minimal required efficacy of the drug that is required to treat, consequently there is also a minimal required dose. For the drug to be therapeutically useful, this minimal required dose needs to be lower than the maximal allowed dose (with respect to the liver function).

Experimental models have been used for more than a century as surrogates, under the assumption that they constitute useful predictors of human metabolism. Now that the Patlak model has been extended to include the dose-dependent effect of a drug (Fig 6A), it can, at least in principle, be used to predict how liver function (gadoxetate uptake) would be affected in humans, also under conditions that for a number of reasons may be difficult or even impossible to test in humans. It is important to note that in the following we have assumed that dose-response of the drug is the same in both humans and rats, *which it is not*. Therefore, the analysis here is an illustration of how translation could be implemented, when metabolic species differences have been both characterized and reconciled. Fig 6C and 6E shows examples of three dose-response curves for three patients (P1-P3), taken from our cohort [11], each having different measured gadoxetate uptake rates without the drug. As can be seen, P1 has a much higher uptake rate (4.1 ms^-1^) than P2 (2.5 ms^-1^) and P3 (1.4 ms^-1^) (1B Column 2). Using the dose response curve already obtained (Fig 4), we can plot how these uptake rates would decrease with increasing drug doses (Fig 6C and 6E). These drug effects can be compared with the normal ranges of uptake rates. These ranges are taken from Forsgren et al. [11] (Fig 6D), who calculated the gadoxetate uptake rate in patients with and without liver fibrosis. The dose where the uptake rate falls below the normal limit, seen in patients without fibrosis, can be considered as the maximal allowed dose (Fig 6C and 6E; Table 1B (Column 3). In other words, that dose is the highest dose that can be given before liver function is compromised beyond normal levels. This maximal allowed dose of the drug should then be compared to the minimal required dose, i.e., the lowest dose that must be given for the drug to have its required efficacy. Fig 6F shows the shape of a hypothetical dose-efficacy curve for our studied drug (Table 1B Column 4). A drug should only be given if the minimal required dose is smaller than the maximal allowed dose, and only in that dosing range. For our studied drug and the three patients, P1 and P2 satisfy these conditions, whereas P3 need to take another drug (Table 1B Column 5). Last, Fig 6B shows how the uptake of gadoxetate would change if one of the human patients (P1) would be given the same drug as the rats at a range of different doses.

## Discussion

We present the first translational model-based framework using DCE-MRI for predicting drug-modulated liver function in humans. In this, we first identified the ‘Patlak model’ as the optimal choice, since it could explain all available human (Fig 2) and rat (Fig 3) data, and since the identifiability analysis showed that the Patlak model consistently provided the most well-defined parameters (Fig 4). The Patlak model in combination with data from rats were then used to establish a new NLME-based model for determining the effects of a hepatotoxic drug on the gadoxetate pharmacokinetic parameters (Fig 5). Finally, we illustrated how the framework in future applications can be used for drug dosage selection, including how it in principle can be applied in personalized treatments designed to avoid DILI (Fig 6).

It is encouraging that all three pharmacokinetic models used here were capable of fitting the human data and that the estimated uptake rates were similar (Fig 2). The fact that the three models provided strikingly similar uptake rates, even though they were developed using completely different approaches, which validates the pharmacokinetic modeling approach for measuring physiological parameters. In other words, this indicates that we are not just measuring model-specific parameters, but actual physiological uptake rates. However, our results also show that one needs to be consistent with the choice of input signal in the models, *i*.*e*., only spleen or blood should be used as an input, to allow for consistency. If the same inputs are used, the results agree well (Fig 2D–2F; R>0.77; close to the unity line), but with different inputs, the agreements are worse (Fig 2B and 2C; R<0.51; away from the unity line).

### On the choice of imaging protocol

In this work, we acquired human images using breath-hold and a protocol that allowed for high spatial resolution. This imaging protocol had the advantage that it resulted in an image quality that matched those obtained in clinical routine liver MRI-protocols [11]; in other words, it would be possible to easily include the proposed translational framework *in a clinical workflow*. On the other hand, a disadvantage with (and a consequence of) our choice of imaging protocol is that the temporal resolution of the data is relatively sparse. This low temporal resolution could potentially influence the results, especially for the Perfusion model [20], see discussion below, which was specifically designed for acquiring high temporal resolution data. However, the results in Fig 2E and 2F indicates that the low temporal resolution does not systematically bias the estimated uptake rate. Nevertheless, data with higher temporal resolution might have identified another model as the optimal choice with respect to parameter identifiability.

### On the choice of modelling approach

Several different modelling approaches have been introduced previously. The first approach (Fig 1C; Patlak model) uses simple two-compartment model. The Patlak model estimates the gadoxetate uptake and efflux rates and uses an input function sampled in the blood [21, 22], or spleen [12, 23]. The model then assumes that the blood extracellular concentration in the liver is the same as the concentration in the blood, which is the same thing as assuming that the mean transit time and the contrast agent in the extracellular space is considered negligible. The Patlak model is therefore typically used when there is not enough temporal resolution in the data to properly characterize the blood flow [21, 22], *e*.*g*. when using images from standard clinical gadoxetate-enhanced MRI-protocols. Such images are typically acquired during breath-hold with enough spatial resolution to be used for radiological reading.

The second approach (Fig 1C; Perfusion model) is based on perfusion MRI, which typically requires measurements of blood flow, and modelling of DCE-MRI data [24]. For DCE-MRI with gadoxetate, such models can relatively easily be modified to also include the hepatic uptake rate, and then a dual-input two-compartment uptake model is created. This a model can simultaneously quantify both liver perfusion and liver function [25]. The perfusion model uses an impulse response, which is convolved with input data from the blood, sampled in both the aorta and portal vein (hence the dual-input name), to reflect the livers dual blood supply. Later the perfusion model has also been extended to include gadoxetate excretion [20] and transport back to the blood [26]. Furthermore, perfusion approaches typically require a high temporal resolution of the DCE-MRI data in order to be able to characterize the blood flow [24], and is essentially a case of the Patlak model without the assumption of the gadoxetate extracellular transit time being negligible.

A third approach (Fig 1C; ’whole-body model’) is to use a mathematical model that is not based on convolution with an input function [16]. This third model instead simulates coupled ordinary differential equations, describing the distribution of gadoxetate in selected compartments of the ’whole body’, consisting of the whole-body extravascular and extracellular compartment. For simplicity, we refer to this as the ’whole-body model’ (although it does not explicitly suggest that all organs are included in the model). The measured outputs used to quantify the whole-body model are simulated gadoxetate concentrations in the liver and spleen [11]. As the spleen is perfused profusely, in combination with a lack of gadoxetate accumulation, the spleen can be used as a surrogate for measurements of blood. In this way, measurements from the spleen can be used input to the Whole-Body model.

There are detailed models for use with data from liver perfusion, *e*.*g*. for describing uptake kinetics in first passage of blood circulation [27, 28]. Such models could in principle be used to improve the ’whole-body model’ which was used here, although they describe other functional aspects of the liver. However, such model expansions will likely alter the model comparison results herein, since the simplest model was found to be the most optional, due to the unavoidable loss of identifiability in more complex models.

Gadoxetate pharmacokinetics has also been studied pre-clinically in rats, using different approaches in terms of both model structures and types of data. Ulloa *et al*. used the Patlak model, with spleen input, to study the effects of different doses of a hepatotoxic drug [12], and this approach was later applied in a multi-center study [23]. A perfusion model with input from the portal vein was implemented by Giraudeau *et al*. to data from fibrotic rats [29]. Similarly, Georgiou *et al*. studied the effects of DILI [30]. However, the temporal resolution of their procedure was not high to allow for to allow for quantification of blood flow, and the Patlak model was therefore used instead. Scotcher *et al*. has developed a physiologically based pharmacokinetic model for pre-clinical data [31], which is similar in complexity to the Forsgren *et al*.*‘*whole-body model’. Yet other studies have devised models, comparable to the Patlak and Perfusion models, in order to obtain hepatobiliary rates in animal models [32, 33]. Similar to our results, the smaller models (*cf*. Patlak model), was shown to describe data well, yielding the smallest parameter uncertainty. Moreover, in a study by Tadimalla *et al*. [33] the influx vs. efflux results were analogous to our results (see also Fig 4 and S1 Fig). In the end this is interesting since the most reasonable model was the same both for human data, and rat data. This suggests that more elaborate models also require more detailed data, in particular to account for species specific differences. *Ex vivo* studies such as the one by Ziemian *et al*. [34], could potentially aid future model comparison.

Finally, since liver is involved in clearance of drugs, many pharmacometric NLME models describe different detailed aspects of liver clearance [35], and how it is altered in different stages of liver damage [36, 37]. However, these models have not yet been developed to analyze uptake of gadoxetate uptake measured using DCE-MRI.

One goal here was to develop experimentally validated models for a variety of specific liver functions, with the long-term goal of a scalable model ranging from intracellular biochemistry to the whole organ. Nevertheless, obviously no complete model for the organ exists presently.

### On parameter uncertainty

If DCE-MRI with gadoxetate is going to be a useful and meaningful biomarker for liver function and DILI, the uncertainty of the pharmacokinetic parameters should ideally be known and preferably as small as possible. If the uncertainty of the parameters is not known, it will be difficult to determine whether a change in the biomarker for a patient is due to an actual change in the underlying physiology, or just due to experimental variations. However, previously there has been little investigation into the uncertainty of the estimated pharmacokinetic parameters for the described models. Georgiou *et al*. did investigate the test-retest reproducibility [20] with the perfusion model, showing a good reproducibility for the hepatic uptake rate. However, they only used relatively a small number of healthy volunteers. Moreover, a drawback with the test-retest approach to measuring uncertainty is that it requires repeated injections of the gadolinium-containing contrast agents in the subjects. We have previously employed an identifiability analysis for the whole-body model, using another method called profile likelihood [16], although this study was limited by its relatively small sample size. In a follow-up study [11], we extended the study by investigation robustness to missing data points and also shorter data acquisitions protocols, and how the procedure can be improved using nonlinear mixed-effects modelling (NLME) [18].

Most of the models mentioned above have never been investigated with respect to parameter uncertainty, especially not when considering individual patients, and this analysis has not been used as a basis for choosing the most appropriate model. Here we have chosen to focus our uncertainty analysis on three different mathematical models for the liver; these have been developed for the purpose of analyzing DCE-MRI data. And in view of the above discussion, the models were selected because of their demonstrated ability to analyze gadoxetate-based DCE-MRI data, in either rats or humans, and we believe that it is unlikely that other models would be performing better.

### On translational aspects and implications on drug safety

With respect to translational aspects, our results clearly show that the gadoxetate pharmacokinetics not surprisingly is much faster in rats compared to humans. This is evident both qualitatively, by visual inspection of the time series, and also from the quantitative values of the uptake rates. In healthy rats, the gadoxetate concentration reached a maximum within just a few minutes after injection (Fig 3A) and the estimated uptake rates were in the range of 1–3*10^−2^ s^-1^ (Fig 4). In humans on the other hand, the gadoxetate concentration approached a plateau after 30 minutes (Fig 2A) and the determined uptake rates were in the range of 0–4*10^−3^ s^-1^. In other words, the two species differed by almost an order of magnitude. These results were broadly anticipated, based on several reports on the uptake rates of OATP-substrates in rat liver versus the corresponding observations in humans [38–40]. One explanation for such a large difference could be different affinities for gadoxetate in humans and rats, since the OATP1B1/B3 transporters are not represented by undisputed orthologs in rats [38, 41]. Another explanation is that there can be a difference in the amounts of active transporters expressed in the hepatocytes [42]. Such differences could be problematic, especially if one is investigating drug-drug interactions and interpreting how drugs interact with specific transporters [38]. One possible way to minimize this problem could be to use knock-in animals, that express the actual human OATP1 variants [43]. Another approach to investigate these differences would be to apply each drug of interest to both human and rat hepatocytes *in vitro*.

Finally, a few concluding remarks on translation aspects with respect to our present work. Future work should include a suitable range of validation procedures in order to test the strengths of our predictions. Ideally such validation should include trials on humans, which could be done by considering a drug that is already used in humans and where corresponding rat data is available. This was not the case for the drug studied herein, as it was part of a drug development project that did not reach the level for human trials. The identified drug must be safe enough for usage in humans, but still must impact liver function to a detectable extent. Note that cellular studies alone will not be sufficient in this context, as they will neither provide information on *in situ* nor *in vivo* conditions.

This work does not describe a complete approach on how to examine the safety of a drug; it is rather a *proof-of-concept* on how to device a model to explore the effects of a drug on liver function. Also, the specific choice of the drug used here is in itself and in principle not important for the conclusions, as our framework will translate all types of liver injuries into quantitative gadoxetate uptake rates, independent of the exact molecular action of the drug. This is a limitation, but the transport processes nevertheless represent a central core function of the liver, and we believe that it would likely be affected by a range of significant changes in liver status.

### On limitations of this work

Our choice of imaging protocol may contribute to certain issues with the input signal. Measuring gadoxetate concentration in the portal vein and aorta can potentially be confounded by image artefacts originating from the blood flow [44]. Moreover, it is much more challenging, and also operator dependent, to position regions of interest (ROIs) within the blood vessels, especially in the portal vein, as the portal vein is much smaller and less well -resolved than the spleen; this may contribute to additional variance obtained in the measurements. In contrast, artefacts of this kind are not a problem in the spleen. Such difference is demonstrated by the lower correlation when blood vessels are used for input signal (Fig 2B and 2C; R<0.51), compared to when the spleen is used (Fig 2D–2F; R>0.77).

While we believe that out new methods could be useful in certain cases, there are also cases where it probably should not be implemented. We believe that the framework may be of significant value for evaluating drug safety and toxicity in the drug development process. More specifically, the framework can be used to translate results from preclinical rat models to humans in order to better estimate if, and to which extent, a certain drug might affect human liver function. With respect to personalized medicine, it may not be the best use of clinical resources to use the approach in all patient groups, due to the high cost of both MR-examinations and the hepatocyte specific contrast agent. The proposed methods may be most useful in selected patient groups, where one suspects that DILI may be a problem, either because the drug is known to be especially hepatotoxic, or because a patient is suspected to have existing liver problems.

### Future aspects of translational work

In addition, there are a few other aspects of our proposed framework that would be interesting to examine, but which have been out of scope for this work. First, we assumed that the drug *interacts* with human hepatocytes in the same way as the rat hepatocytes, and this may, or may not, be a limitation of our approach. In future applications, this should be investigated by suitably devised *in-vitro* studies. Moreover, we assumed that the *pharmacokinetics* of the drug were the same in rats and humans. Again, this may not be an entirely valid assumption, although such conceptual pharmacokinetic translations of drug actions are nevertheless commonly used in early drug development pipelines. In our view it is very important in translational medicine to examine the translation of pharmacodynamic properties, including the hepatotoxic effects of a drug, however, this needs additional work, and our present work is just a contribution to meet this important need.

## Supporting information

S1 Fig Likelihood profiles for the parameter k_eff_ corresponding to the parameter estimation to the 35 individual patient datasets presented in Fig 4 in the main text. As can be seen in the likelihood profiles for the Prefusion model (yellow lines), for a lot of patients no lower limit for the k_ef_ parameter can be found (lower limit can be found in 10 out 35 patients). For the Patlak model (blue lines), a lower limit can be found in 20 out of 35 patients. In contrast, for the Whole-body level (green lines), a lower-limit of k_ef_ was not found for any patient. The allowed lower-limit in parameter range was the same for the Patlak and the Perfusion (10^−8^), while the Whole-body had a allowed lower limit at 10^−5^.
(TIF)

S2 Fig Quantitative analysis of the uptake rate k_i_ parameter uncertainty confidence intervals (CI), also denoted likelihood profiles, shown in Fig 4 in the main text. **A)** Comparing the overlap of the parameter uncertainty CI for the Patlak model with the Perfusion, and Whole-body model. Looking at first comparison, in 91.4% of all presented CI the Patlak model parameters is a subset of the Prefusion model parameter CI. For the second comparison, in similar fashion, 94.3% of all Patlak CI are a subset of the Whole-body CI. **B)** Figure showing the mean CI length for all fitted data series, for the three different models. As can be seen, the Patlak model CI length is significantly smaller compared to the other two models.
(TIF)

## Abbreviation

DILI: drug induced liver injury
