# Supplementary figures and images for: Mathematical models for biomarker calculation of drug-induced liver injury in humans and experimental models based on gadoxetate enhanced magnetic resonance imaging
Source: PLoS One. 2023 Jan 6;18(1):e0279168. doi: 10.1371/journal.pone.0279168 (PMC9821424; doi:10.1371/journal.pone.0279168)

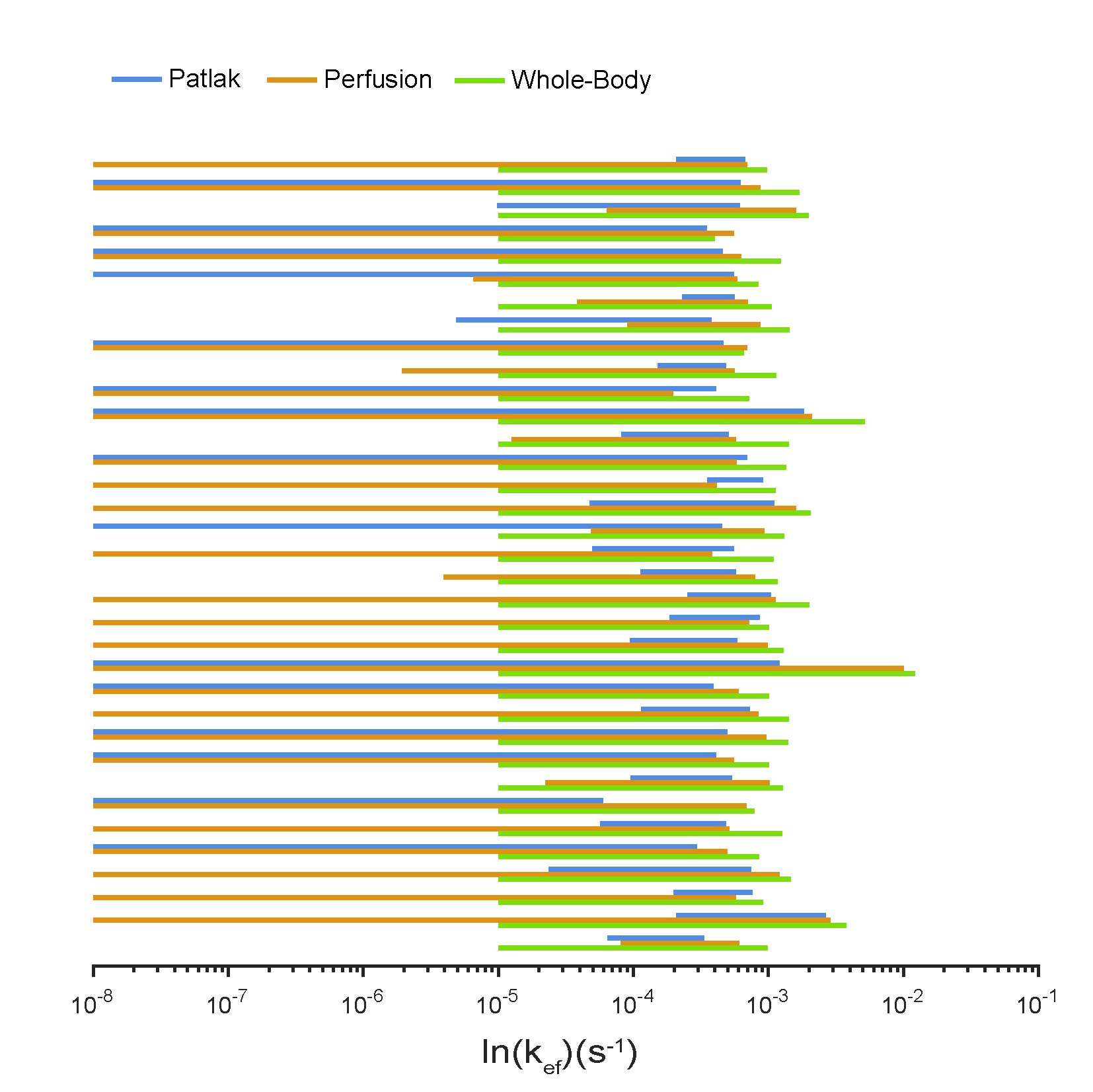

Supplement: S1 Fig — As can be seen in the likelihood profiles for the Prefusion model (yellow lines), for a lot of patients no lower limit for the kef parameter can be found (lower limit can be found in 10 out 35 patients). For the Patlak model (blue lines), a lower limit can be found in 20 out of 35 patients. In contrast, for the Whole-body level (green lines), a lower-limit of kef was not found for any patient. The allowed lower-limit in parameter range was the same for the Patlak and the Perfusion (10−8), while the Whole-body had a allowed lower limit at 10−5. (TIF) [file pone.0279168.s001.tif]

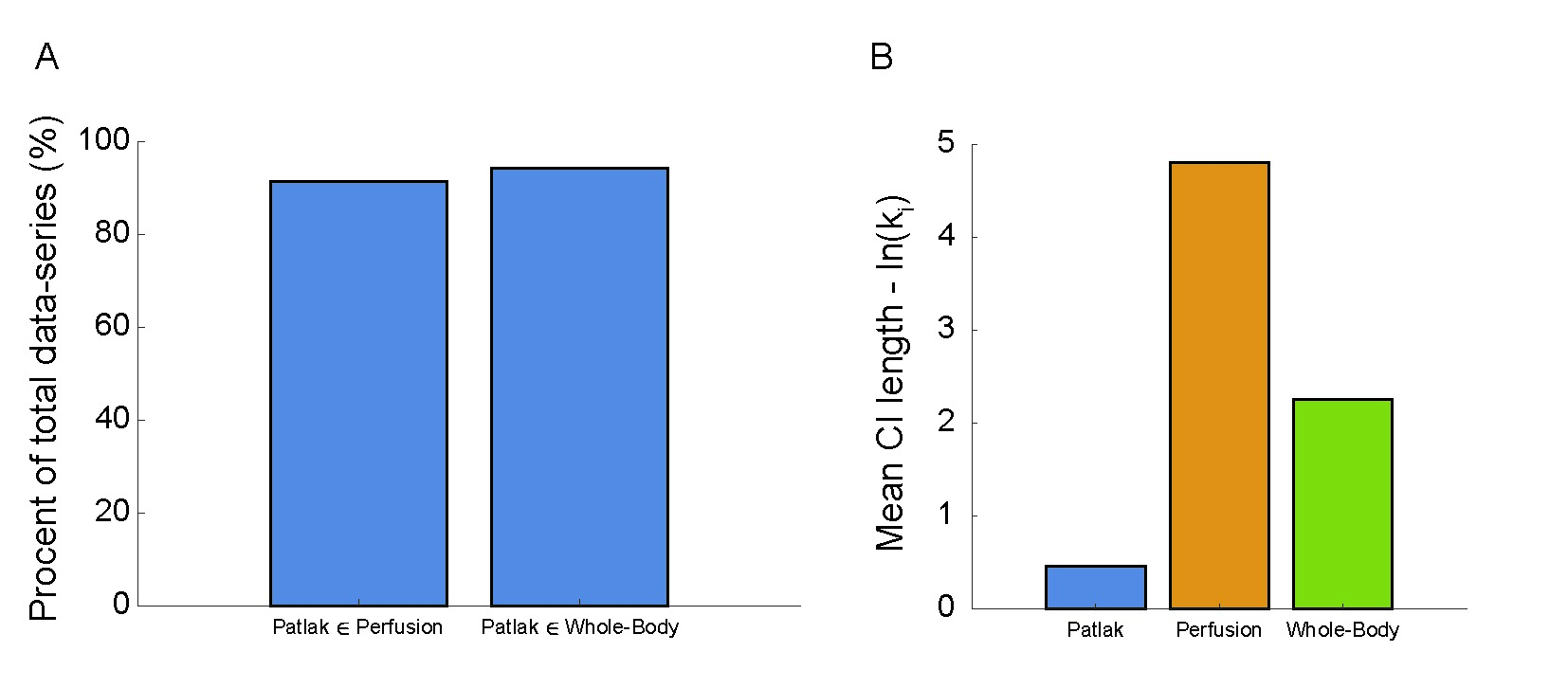

Supplement: S2 Fig — A) Comparing the overlap of the parameter uncertainty CI for the Patlak model with the Perfusion, and Whole-body model. Looking at first comparison, in 91.4% of all presented CI the Patlak model parameters is a subset of the Prefusion model parameter CI. For the second comparison, in similar fashion, 94.3% of all Patlak CI are a subset of the Whole-body CI. B) Figure showing the mean CI length for all fitted data series, for the three different models. As can be seen, the Patlak model CI length is significantly smaller compared to the other two models. (TIF) [file pone.0279168.s002.tif]
